# NSC23925, Identified in a High-Throughput Cell-Based Screen, Reverses Multidrug Resistance

**DOI:** 10.1371/journal.pone.0007415

**Published:** 2009-10-12

**Authors:** Zhenfeng Duan, Edwin Choy, Francis J. Hornicek

**Affiliations:** Sarcoma Biology Laboratory, Center for Sarcoma and Connective Tissue Oncology, Massachusetts General Hospital, Boston, Massachusetts, United States of America; Bauer Research Foundation, United States of America

## Abstract

**Background:**

Multidrug resistance (MDR) is a major factor which contributes to the failure of cancer chemotherapy, and numerous efforts have been attempted to overcome MDR. To date, none of these attempts have yielded a tolerable and effective therapy to reverse MDR; thus, identification of new agents would be useful both clinically and scientifically.

**Methodology/Principal Findings:**

To identify small molecule compounds that can reverse chemoresistance, we developed a 96-well plate high-throughput cell-based screening assay in a paclitaxel resistant ovarian cancer cell line. Coincubating cells with a sublethal concentration of paclitaxel in combination with each of 2,000 small molecule compounds from the National Cancer Institute Diversity Set Library, we identified a previously uncharacterized molecule, NSC23925, that inhibits Pgp1 and reverses MDR1 (Pgp1) but does not inhibit MRP or BCRP-mediated MDR. The cytotoxic activity of NSC23925 was further evaluated using a panel of cancer cell lines expressing Pgp1, MRP, and BCRP. We found that at a concentration of >10 µM NSC23925 moderately inhibits the proliferation of both sensitive and resistant cell lines with almost equal activity, but its inhibitory effect was not altered by co-incubation with the Pgp1 inhibitor, verapamil, suggesting that NSC23925 itself is not a substrate of Pgp1. Additionally, NSC23925 increases the intracellular accumulation of Pgp1 substrates: calcein AM, Rhodamine-123, paclitaxel, mitoxantrone, and doxorubicin. Interestingly, we further observed that, although NSC23925 directly inhibits the function of Pgp1 in a dose-dependent manner without altering the total expression level of Pgp1, NSC23925 actually stimulates ATPase activity of Pgp, a phenomenon seen in other Pgp inhibitors.

**Conclusions/Significance:**

The ability of NSC23925 to restore sensitivity to the cytotoxic effects of chemotherapy or to prevent resistance could significantly benefit cancer patients.

## Introduction

Patients with advanced cancer are usually treated with chemotherapy in order to extend life and palliate symptoms. There is some improvement of responses seen in select cases by the addition of immunotherapy or biological response modifiers [1]. Despite considerable advances in anticancer drug development, with few exceptions, metastatic solid tumors in adults remain largely incurable in part because of their eventual development of resistance to chemotherapy [2]. The inevitable development of multidrug resistant cancer cells during the course of treatment is a major fundamental obstacle associated with cancer care. In these cases, cancer cells become resistant to a variety of anticancer drugs with different structures and mechanisms of action even if the patient's cancer has not been previously exposed to a particular drug - a phenomenon known as cross-resistance. One type of cross resistance known as multidrug resistance (MDR) has been studied widely [2], [3]. Other mechanisms of cross-resistance to chemotherapy include alterations in pharmacokinetics, poor drug penetration through the extracellular matrix, cell adhesion and increased intratumoral hydrostatic pressure [3], [4], [5], [6]. Both intrinsic and acquired cellular drug resistance has been extensively studied using cell lines induced to be resistant to chemotherapeutic drugs' cytotoxic effects by prolonged culture in gradually increasing levels of cytotoxic agents [7], [8], [9]. Although there are several different intrinsic cellular mechanisms associated with the development of MDR, an important molecular basis for MDR is overexpression of plasma membrane glycoprotein (Pgp1) [3], [10]. Pgp1 is the best known mediator of MDR. Pgp1 is the gene product of MDR1(ABCB1), a member of the ABC (ATP binding cassette) superfamily of transporter proteins, and it acts as an energy-dependent cancer drug efflux pump, preventing adequate intracellular accumulation of a large number of cytotoxic drugs including anthracyclines (doxorubicin,daunorubicin), vinca alkaloids (vinblastine), taxanes (paclitaxel, docetaxel) and many others. Innate or acquired expression of Pgp1 is therefore a major problem in the treatment of patients with many types of cancer [3], [5].

Pgp1 (MDR1) expression, frequently detected in both solid and hematologic malignancies, as well as in cancer stem cells, is a marker of chemoresistance and decreased survival in leukemia, lymphoma, osteosarcoma, small-cell lung cancer, ovarian cancer, breast cancer, and other malignancies [2], [3], [4]. Although current chemotherapy regimens can achieve responses in patients with solid tumors, recurrences are typical. When recurrent, tumors often have acquired MDR, either by adaptation of previously Pgp1-negative tumor cells or by selection for drug-resistant Pgp1 positive tumor cells [2], [3]. Elimination of MDR cells during initial treatment or at the time of recurrence is necessary to achieve greater effect from chemotherapy.

A wide range of compounds that interact with Pgp1 and block drug efflux have been reported to reverse the MDR phenotype [10], [11]. Unfortunately, these compounds are relatively nonspecific and have low potency at doses patients can tolerate. With the majority of these compounds, clinical toxicities associated with their use at concentrations required to inhibit Pgp1 have precluded their widespread use [10], [12]. For example, verapamil, a calcium channel blocker that also inhibits Pgp1, is associated with cardiac toxicity at doses required to reverse MDR [13], [14], [15]. Full reversion of MDR by verapamil requires a concentration of approximately 10 µM in most cell culture models; whereas plasma levels above 1 µM results in complete heart block [10], [16], [17]. Cyclosporin A (CsA), besides reversing Pgp1, is also used clinically for immunosuppression. However, at doses used to reverse MDR, the immunosuppressive effect of CsA is harmful and, at higher concentrations, CsA is nephrotoxic [10], [17], [18], [19]. Another MDR reversing agent, PSC833, has been reported to induce cerebellar ataxia and hyperbilirubinemia [20], [21]. Although CsA and PSC 833 both inhibit Pgp1, they have some modest inhibition of MRP1 [3], [10]. The results from PSC 833 in phase III clinical trials showed no survival benefits, consistent with of the other Pgp1 inhibitors studied up to now [10], [11]. The development of potent and selective MDR inhibitors is an important unmet need for cancer patients in the clinic.

Screening of chemical libraries for biologically active compounds aimed at certain cellular targets is a powerful approach to identify small molecules regulating cellular function that can be useful as both research tools and therapeutic agents [22], [23], [24]. We screened 2000 small molecule compounds from the NCI Diversity Set library to look for compounds that can reverse MDR in a drug-resistant cell line. We observed that NSC23925 was the most potent MDR reversing compound in this set. We showed that NSC23925 can reverse drug-resistance for other MDR cell lines, including ovarian, breast, colon cancer, and sarcoma cell lines. NSC23925 was able to resensitize all of these cell lines to paclitaxel, doxorubicin, vincristine, ET-743 and PM00104. We anticipate that NSC23925 or a derivative of this compound may have therapeutic value in the treatment of a variety of MDR dependent cancers.

## Materials and Methods

### Drugs and chemicals

The Structural Diversity Set is a library of approximately 2,000 small molecules derived from the almost 140,000 compounds available on plates through the National Cancer Institute (NCI). These compounds are thought to have anti-tumor activity but their functional data is limited. Detailed information on the selection, structures, and activities of these diversity set compounds can be found on the NCI Developmental Therapeutics Program web site (http://dtp.nci.nih.gov). Paclitaxel, doxorubicin, and cisplatin were obtained through unused residual clinical material provided by the pharmacy at the Massachusetts General Hospital. ET-743 (trabectidin, Yondelis) and PM00104 (Zalypsis) were supplied by PharmaMar (Spain).

### Cell Lines and antibodies

The paclitaxel-resistant U-2OS_TR_, SKOV-3_TR_, OVCAR8_TR_, MCF-7_TR_ and SW480_TR_ lines as well as doxorubicin resistant MCF-7_DR_ and gemcitbine resistant OVCAR5_GR_ cell lines were established as previously reported [7], [8], [9], [16]. The osteosarcoma cell line U-2OS, KHOS, uterine sarcoma cell line MESSA and its doxorubicin selected drug resistant cell line MESSA/Dx5, non-small cell lung cancer cell line H-69 and it's doxorubicin selected drug resistant cell line H-69AR (overexpress MRP1) were obtained from the ATCC (Rockville, MD). Dr. Efstathios Gonos (Institute of Biological Research & Biotechnology, Athens, Greece) provided the doxorubicin resistant U-2OS R2 (referred in the text below as U-2OS_DR_), KHOS R2 cell lines [25]. Dr. Stephen. Howell (The University of California Medical Center, San Diego) provided the cisplatin-resistant ovarian cancer IGROV1cp and 2008cp70 cell lines. Dr. Erasmus Schneider (Wadsworth Center, Albany) provided the mitoxantrane resistant breast cancer MCF-7/MX cell line. Dr. Katia Scotlandi (Institute Orthopedics Rizzoli, Italy) provided ET-743 resistant TC-ET 6 nM and TC-ET 12 nM cell lines.

The Pgp1 monoclonal antibody was purchased from Signet (Dedham, MA). The monoclonal antibody to MRP1 and MTT reagent were purchased from Sigma-Aldrich (St. Louis, MO). The monoclonal antibody to BCRP antibody was purchased from Chemicon (Temecula, CA).

### Cell culture

All the cell lines were cultured in RPMI 1640 (Invitrogen, Carlsbad, CA) supplemented with 10% fetal bovine serum, 100-units/ml penicillin and 100 µg/ml streptomycin (Invitrogen). Cells were incubated at 37°C in 5% CO_2_-95% air atmosphere and passaged when near confluent monolayers were achieved using 2% trypsin-EDTA solution. Drug-resistant cell lines were periodically cultured in the respective drug to confirm their drug resistance characteristics.

### High-throughput drug cytotoxicity assay

**Figure 1:**
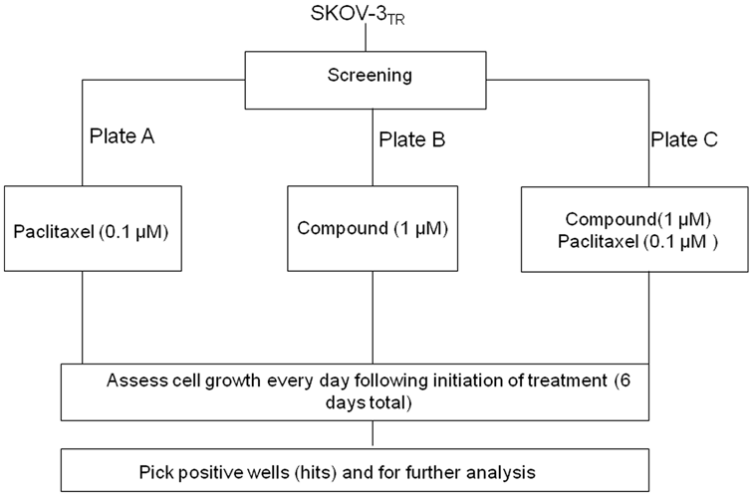
Flowchart of the screening small molecular compound to reverse drug resistance. For the screen, 2×10^4^ SKOV-3_TR_ cells per well in 96-well plates were added with different NCI Diversity Set library compound and paclitaxel as indicated. After culturing for 6 days, the number of viable cells was analyzed using the CellTiter 96 AQueous One Solution Cell Cytotoxicity Assay and visualized under the microscope.

SKOV-3_TR_ cells with chemotherapy drug paclitaxel were used for initial high-throughput screening. SKOV-3_TR_ showed 100-fold more resistance to paclitaxel as compared with parental sensitive cells and could grow in 0.3 µM concentration of paclitaxel without showing toxicity. Screening conditions in SKOV-3_TR_ were optimized in 96-well plates for growth conditions, small molecular compound and drug concentration and assay times prior to screening. On day 1, SKOV-3_TR_ cells were seeded at 2×10^4^ cells/well on 96-well plates and labeled with plate A, B, C and incubated for 24 hr at 37°C (Fig. 1). On day 2, plate A was added with 0.1 µM of paclitaxel, plate B was added with different screening small molecular compound at 1 µM to each well without paclitaxel; Plate C was added with both small molecular compound and paclitaxel at the same concentration as described in plate A and B. From day 4 to day 6, fresh medium was replaced with necessity as described above and evaluated for cytotoxicity under the microscope. On Day 6, the number of viable cells was evaluated for cytotoxicity in any wells under the microscope manually and final results were determined via CellTiter 96®AQ_ueous_ One Solution Cell Cytotoxicity Assay (Promega, Madison, WI) by a SPECTRAmax® Microplate Spectrophotometer (Molecular Devices, Sunnyvale, CA). Only the compounds at 1 µM showed no cytotoxicity (without paclitaxel) to SKOV-3_TR_ were selected for further study.

### Determination of the results and analysis

The general format was designed for screening the small molecular compound library and identifying novel drug resistant inhibitor. These include evaluation of each small molecular compound through a well studied multidrug resistant cell line SKOV-3_TR_. The experiments were conducted in two control plates (A and B) and one experimental plate (C) to permit plate-to-plate comparisons (Fig. 1). The control plates (plate A and B) were used to evaluate cytotoxicity of paclitaxel and small molecular compound itself, and to exclude compound that were lethal to the cells in the absence of chemotherapy drug. Plate B was necessary because the goal of this study was to identify small molecular compound that can reverse drug resistance and not to determine cell toxicity. Therefore, one plate (plate B) was given only small molecular compound to confirm that the small molecule is not lethal in the absence of paclitaxel (a positive result is cell survival); Plate C was given both small molecular compound and 0.1 µM paclitaxel. This is typically a sublethal dose of paclitaxel for the SKOV-3_TR_ cell line and a positive result would be cell death at 4–6 days. Small molecular compound that were associated with cell survival in plate A and plate B and death in plate C were identified as “hits” and selected for further study. Once hits were identified, we validated that the hits indeed could reverse drug resistance in other drug resistant cell lines.

### Cytotoxicity assay

Drug cytotoxicity was assessed *in vitro* using the MTT assay as previously described [26], [27]. Drugs at the concentrations utilized in the MTT assay were performed in the absence of cells to verify no change in absorbances. Experiments were performed in triplicate.

### Duration of MDR reversal

The experiment was performed as previously described [28]. In brief, 8×10^5^ SKOV-3_TR_ cells/ml were incubated for 24 h with or without NSC23925 or verapamil before being washed 3 times with growth medium. The cells were then incubated for 4 days with addition of varying concentrations of paclitaxel. Final results were determined by MTT assay as described above.

### Western blotting

Pgp1, MRP1, and BCRP proteins were analyzed in total cell lysates. Total cell lysates were prepared, and Western blot analysis was performed as previously described [8].

### Drug efflux assay

The Vybrant™ multi-drug resistance assay kit (Invitrogen/Molecular Probes) was used to measure drug efflux properties of different resistant cell lines. This assay utilizes the fluorogenic dye calcein acetoxymethyl ester (calcein AM) as a substrate for efflux activity of Pgp1 or other membrane pump ABC proteins. Calcein AM is taken up by cells and hydrolyzed by cytoplasmic esterases to fluorescent calcein. Calcein AM is well retained in the cytosol. However, multidrug resistant cells expressing high levels of Pgp1 rapidly extrude non-fluorescent calcein AM from the plasma membrane, reducing accumulation of fluorescent calcein in the cytosol. Drug sensitive and resistant cells (50,000 cells per well) were cultured in 96-well plate for 24 hours. Triplicated cultures of cells were treated with NSC23925, verapamil for one hour and then incubated in calcein AM in 100 µl total volume. After 30 minutes, the cells in plate were washed and centrifuged twice with 200 µl cold RPMI1640 culture medium, and cell fluorescence was measured at a wavelength of 490 nm (A_490_) on a SPECTRAmax® Microplate Spectrofluorometer (Molecular Devices).

### Fluorescence microscopy

For visualization of the effects of NSC23925 on the intracellular retention of calcein AM, doxorubicin, and mitoxantrone, 10,000 resistant cells were seeded on to Lab-Tek 8-well chamber slides on the day prior to the assay. Cells were then incubated with either, 0.25 µM calcein AM, 10 µM doxorubicin, or 10 µM mitoxantrone either alone or in the presence of NSC23925 in RPMI 1640 media for one hour at 37°C. Images were acquired by Nikon Eclipse Ti-U fluorescence microscope (Nikon Corp.) equipped with a SPOT RT digital camera (Diagnostic Instruments, Inc., Sterling Heights, MI).

### PGP-ATPase assay

The Pgp-Glo™ Assay Systems (Promega) provide the necessary reagents for performing luminescent P-glycoprotein (Pgp1) ATPase assays. PGP-ATPase assay is a valuable screening tool for determining if a drug interacts with Pgp1. The ATPase assay provides a rapid, colorimetric, compound-independent measure of the concentration-dependence of any interaction of a drug with Ppg1. If a drug does not stimulate Pgp1-ATPase activity, the assay can still determine if the drug interacts with Pgp1 as an inhibitor of the ATPase activity stimulated by a known substrate, such as verapamil. The effect of NSC23925 on the ATPase activity of Pgp1 was measured according to the manufacturer's protocol.

### Statistical data analysis

Values shows are representative of triplicate determinations in two or more experiments. Treatment effects were evaluated using a two-sided Student's t test (GraphPadPRISM® 4 software, GraphPad Software, San Diego, CA). Errors are SD of averaged results and P<0.05 values were accepted as a significant difference between means.

## Results

### Establishment of cell based high-throughput screening of drug resistance inhibitors

To identify candidate small molecule compounds that can reverse drug resistance in MDR cell lines, we designed an assay to detect reconstitution of paclitaxel cytotoxicity in a Pgp1-expressing, multidrug resistant human ovarian cancer cell line SKOV-3_TR_. 0.1 µM paclitaxel was determined to be the concentration of paclitaxel that is lethal to drug sensitive SKOV-3 cells, but does not affect the growth of drug resistant SKOV-3_TR_ cells. Cells were arrayed in 96-well plates and each well was incubated for six days in optimized culture conditions with 0.1 µM paclitaxel, 1 µM library compound, or both 0.1 µM paclitaxel and 1 µM library compound (Fig. 1). Cell growth was assessed daily for six days (see Materials and Methods section).

### Identification of small molecule compound, NSC23925, as a small molecule compound that reverses multiple drug resistance in human cancer cell lines

**Figure 2:**
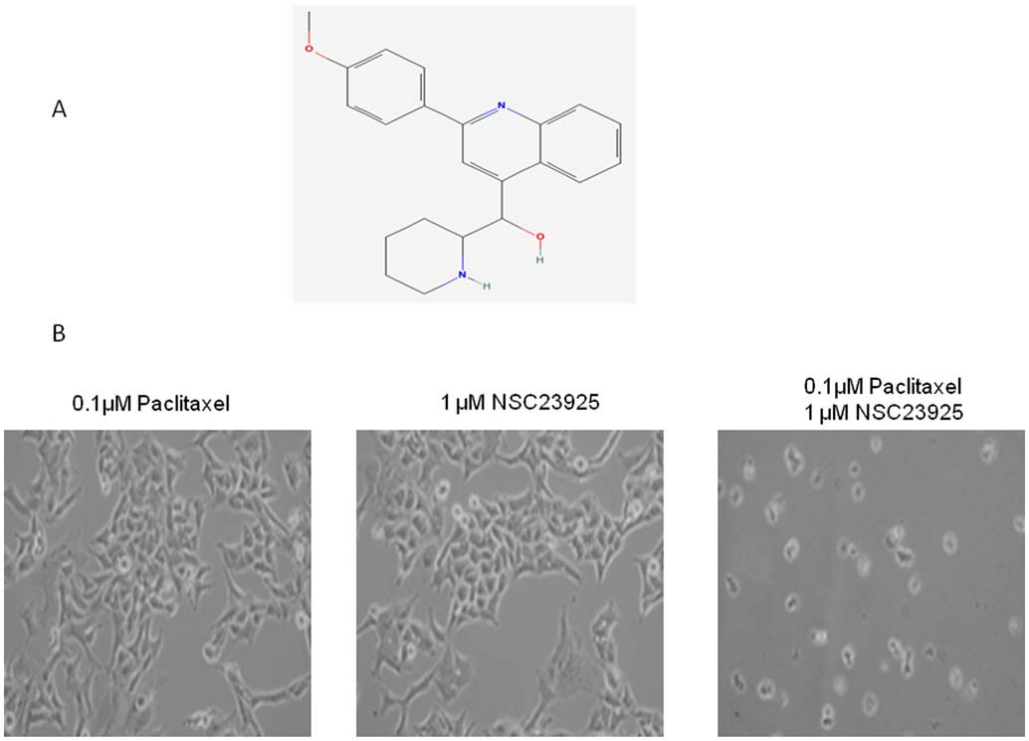
Chemical structure of NSC23925 and reverse paclitaxel resistance by NSC23925. *A*: Structure of NSC23925. *B*: NSC23925 in combination with 0.1 µM paclitaxel induces significantly cell death in SKOV-3_TR_.

After screening ∼2,000 small molecule compounds in the NCI Diversity Set library for synergistic cytotoxicity with paclitaxel, there were ∼200 compounds that showed cytotoxicity (without paclitaxel) to SKOV-3_TR_ at a concentration of 1 µM. Two compounds, NSC23925 and NSC77037, were identified as the agents with highest activity to reverse drug resistance without cytotoxicity by itself (Fig. 2A). NSC77037(Tetrandrine), a bis-benzylisoquinoline alkaloid compound, has been shown to reverse doxorubicin resistance in a breast cancer cell line both in vitro and in vivo [29], [30], [31]. In contrast, there are no prior reports describing NSC23925 (2-(4-methoxyphenyl)-4-quinolinyl)(2-piperidinyl) methanol) or its activity for reversing drug resistance. When combined with 1 µM NSC23925, 0.1 µM paclitaxel induces significant cell death in SKOV-3_TR_,which grows well in either 0.1 µM paclitaxel or 1 µM NSC23925 alone (Fig. 2B).

### Modulation of drug resistance in MDR cell lines

**Figure 3:**
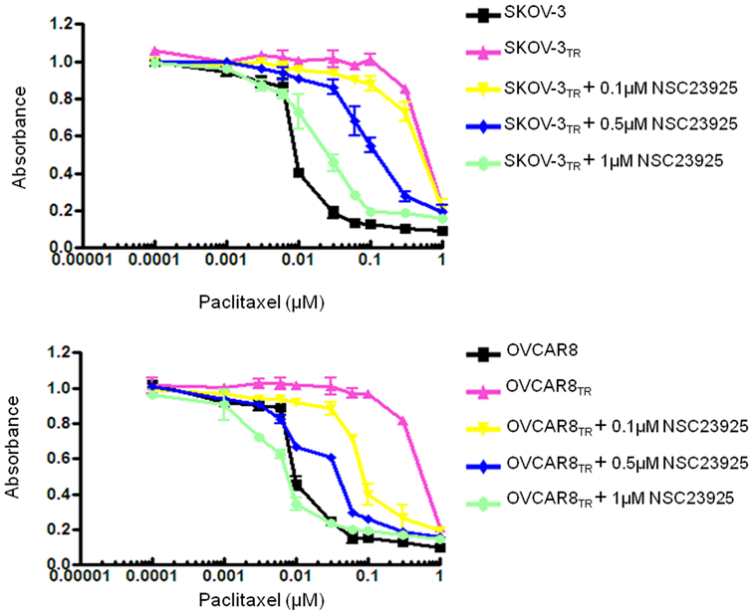
Effect of NSC23925 to reverse drug resistance in MDR cell lines. Cells were treated with the paclitaxel and NSC23925 in RPMI1640 complete media at the indicated concentrations. The relative sensitivity of each line to paclitaxe was determined by MTT analysis 6 days posttreatment. *A*: Reversal of paclitaxel resistance by NSC23925 in SKOV-3_TR_ cells. *B*: Reversal of paclitaxel resistance by NSC23925 in OVCAR8_TR_ cells.

**Figure 4:**
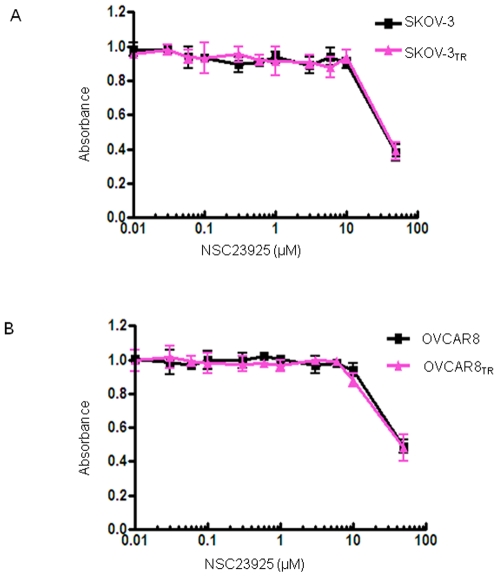
Nonspecific toxicity of NSC23925 on SKOV3 vs SKOV-3_TR_ and OVCAR8 vs OVCAR8_TR_. Cells were treated with the NSC23925 in RPMI1640 complete media at the indicated concentrations. The relative sensitivity of each line to NSC23925 was determined by MTT assay. *A*: Nonspecific toxicity of NSC23925 on SKOV3 vs SKOV-3_TR_ and OVCAR8 vs OVCAR8_TR_. *B*: Nonspecific toxicity of NSC23925 on OVCAR8 vs OVCAR8_TR_.

To determine whether NSC23925 is able to reverse drug resistance in other cancer cell lines, we evaluated the effects of NSC23925 on several MDR cell lines using a variety of anti-cancer agents. We observed that NSC23925 reverses chemoresistance in a wide variety of tumor types where MDR1 is highly expressed; these tumor types include the ovarian cancer cell lines SKOV-3_TR_ and OVCAR8_TR_, breast cancer cell line MCF-7_TR_, and sarcoma cell lines MESSA/Dx5, KHOS R2, and U-2OS_DR_ (representative data from SKOV-3_TR_, OVCAR8_TR_, and KHOS R2 shown in Table 1). Maximal reversal of MDR was typically seen in NSC23925 doses between 0.5 and 1 µM (Fig. 3). NSC23925 was highly active across the panel of cell lines and demonstrated significant reversal of chemoresistance when used in conjunction with paclitaxel, docetaxel, doxorubicin, daunorubicin, gemcitabine, vincristine, ET-743, or PM00104 (Table 2). The potency of NSC23925 was 10 to 50 fold greater than that of verapamil or CsA. Moreover, the presence of <5-10 µM of NSC23925 alone had no cytotoxic effect in the parental cell lines SKOV-3 and OVCAR8 which lack expression of Pgp1 (Fig. 4A and 4B). Importantly, NSC23925 did not alter the cytotoxicity of cisplatin and methotrexate, both agents known to be unaffected by MDR1 mechanisms (Table 2). These results suggest that reversal of resistance was mostly attributable to the inhibition of Pgp1. Furthermore, we tested other cell lines with non-Pgp1 mediated MDR mechanisms (H69/AR cells that express MRP1, but not Pgp1, and MCF-7 MX cells that express BCRP, but not Pgp1) to evaluate the specificity of NSC23925. We observed that NSC23925 was unable to reverse drug resistance in these non-Pgp1 expressing drug resistant cell lines, suggesting that NSC23925 activity is specific for Pgp1.

**Table 1:** Effect of NSC23925 Reversing Drug Resistance in Multidrug Resistant Cell Lines.

	SKOV-3TRIC50 (µM)	OVCAR8TR IC50 (µM)	KHOSR2IC50(µM)
Paclitaxel	0.310±0.021	0.34±0.032	0.21±0.023
Add NSC23925 0.1 µM	0.302±0.056 (1.03)	0.127±0.027 (2.7)	0.16±0.016 (1.3)
Add NSC23925 0.5 µM	0.132±0.047 (2.4)	0.072±0.046 (4.7)	0.12±0.032 (1.8)
Add NSC23925 1 µM	0.037±0.028 (8.4)	0.028±0.047 (12.1)	0.06±0.020 (3.5)
Doxorubicin	22.34±0.2	27.624±0.256	10.34±0.566
Add NSC23925 0.1 µM	16.52±0.21 (1.4)	12.35±0.36 (2.3)	7.364±0.336 (1.4)
Add NSC23925 0.5 µM	6.78±0.16 (3.3)	5.200±0.471 (5.3)	1.205±0.461 (8.6)
Add NSC23925 1 µM	1.94±0.22 (11.5)	1.216±0.122 (23)	0.292±0.210 (35)
ET-743	0.004±0.002	0.006±0.002	0.175±0.025
Add NSC23925 0.1 µM	0.005±0.002 (0.8)	0.005±0.001 (1.2)	0.145±0.045 (1.2)
Add NSC23925 0.5 µM	0.004±0.001 (1)	0.005±0.001 (1.2)	0.083±0.039 (2.1)
Add NSC23925 1 µM	0.004±0.001 (1)	0.007+0.002 (0.9)	0.054±0.034 (3.2)
PM00104	0.006±0.001	0.008±0.002	0.143±0.045
Add NSC23925 0.1 µM	0.005±0.002 (1.2)	0.008±0.002 (1)	0.113±0.038 (1.3)
Add NSC23925 0.5 µM	0.007±0.001 (0.9	0.006±0.002 (1.3)	0.026±0.014 (5.5)
Add NSC23925 1 µM	0.005±0.002 (1.2)	0.005±0.002 (1.6)	0.008±0.016 (17.9)

IC_50_ is the concentration of drug (µM) that produced 50% inhibition of cell growth.

Results were calculated from one experiment with triplicate wells. ± reflect SDEV (SD).

Number in the parentheses represent fold-reversal of drug resistance.

**Table 2:** Reverse Drug Resistance by NSC23925

Decreased drug resistance	No effect
Paclitaxel	Cisplatin
Docetaxel	Carboplatin
Doxorubicin	Topotecan
Vincristine	Methotrexate
Daunorubicin	
Vincristine	
Gemcitabine	
ET-743	
PM00104	

### NSC23925 itself is not a Pgp1 substrate

**Figure 5:**
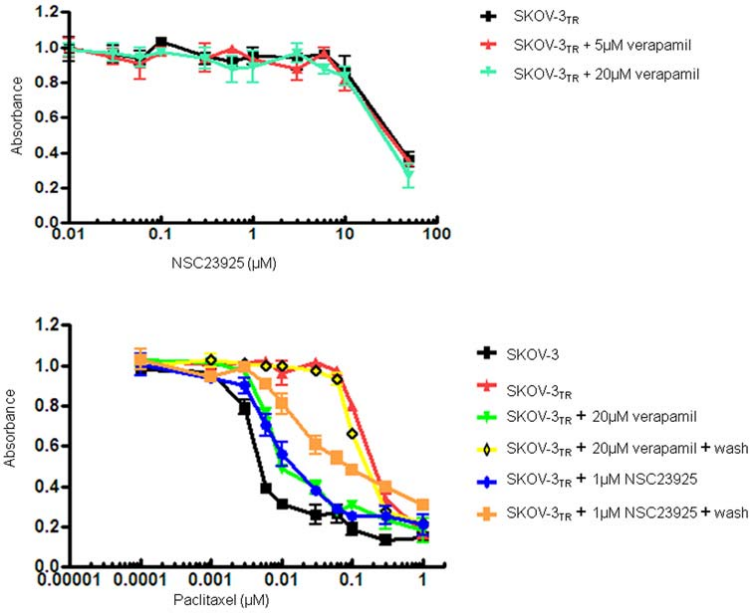
Effect of verapamil on NSC23925 sensitivity in Pgp1-overexpressing cells and persistence of NSC23925 activity. *A*: Effect of verapamil on NSC23925 sensitivity in SKOV-3_TR_ cells. SKOV-3_TR_ cells were treated with different concentration of NSC23925 alone or in combination with verapamil for 72 hours. Drug cytotoxicity was determined by MTT assay as described in the Materials and Methods. *B*: Persistence of NSC23925 reverses paclitaxel resistance in SKOV-3_TR_ cells after incubation and washout of NSC23925 or verapamil.

Although NSC23925 reversed Pgp1 mediated MDR in the nanomolar concentration range, NSC23925 was not cytotoxic by itself at doses up to 10 µM in both sensitive and resistant cell lines. At high concentrations, NSC23925 by itself is equally inhibitory on the proliferation of both SKOV-3 and SKOV-3_TR_ and for OVCAR8 and OVCAR8_TR_ cell lines in a dose-dependent manner (Fig. 4A and 4B). IC_50_s were similar in matched cell lines that did and did not express Pgp1. The IC_50_ for NSC23925 is 8 µM in SKOV-3/SKOV-3_TR_ and 25 µM in OVCAR8/OVCAR8_TR_ cell lines, whereas the mean concentration of NSC23925 required for maximal reversal of resistance in SKOV-3_TR_ or OVCAR8_TR_ to cytotoxic drugs is 0.5 µM to 1 µM (Fig. 3A and 3B). Thus, NSC23925 is cytotoxic at concentrations 10 to 50 fold greater than those required for maximal reversal of drug resistance. This indicates that overexpression of Pgp1 does not confer resistance to the cytotoxic effects of single agent NSC23925. These results also suggest that compounds like NSC23925 are not Pgp1 transport substrates [28], [32], [33]. To confirm this, we determined the effect of the Pgp1 inhibitor, verapamil, on NSC23925 sensitivity in Pgp1-overexpressing SKOV-3_TR_ cells. As expected, verapamil did not influence the cytotoxic effect of NSC23925 in SKOV-3_TR_ cells (Fig. 5A). Similar results were found in OVCAR8_TR_ (data not shown). This data collectively demonstrates that NSC23925 itself is not a substrate of Pgp1, and Pgp1 does not confer resistance to the cytotoxic effects of NSC23925 in tumor cells. Also, NSC23925 is not subject to cross resistance with classical cytotoxic agents such as paclitaxel or doxorubicin. These results are of clinical significance because they suggest that NSC23925 may be a good option for the patients with tumors that have already developed MDR.

### Duration of NSC23925 activity

The ability of NSC23925 to inhibit Pgp1 mediated efflux and its duration of action were investigated using two MDR substrates, paclitaxel, doxorubicin, and two MDR cell lines SKOV-3_TR_ and OVCAR8_TR_. We observed that NSC23925 inhibits the efflux of both paclitaxel and doxorubicin from MDR cells and that the inhibitory effects of NSC23925 remains even when it was washed out from the efflux medium after overnight exposure (1 µM) (Fig. 5B). NSC23925 was effective at inhibiting Pgp1 mediated transport for at least 4 days after wash-out. In comparison, verapamil even after exposure to very high concentration (20 µM), displayed only a short duration (24 h) of inhibition (Fig. 5B). Similar results were obtained in OVCAR8_TR_ cells (data not shown).

### NSC23925 modulates pgp1-mediated uptake and efflux of calcein AM and other Pgp1 substrates

**Figure 6:**
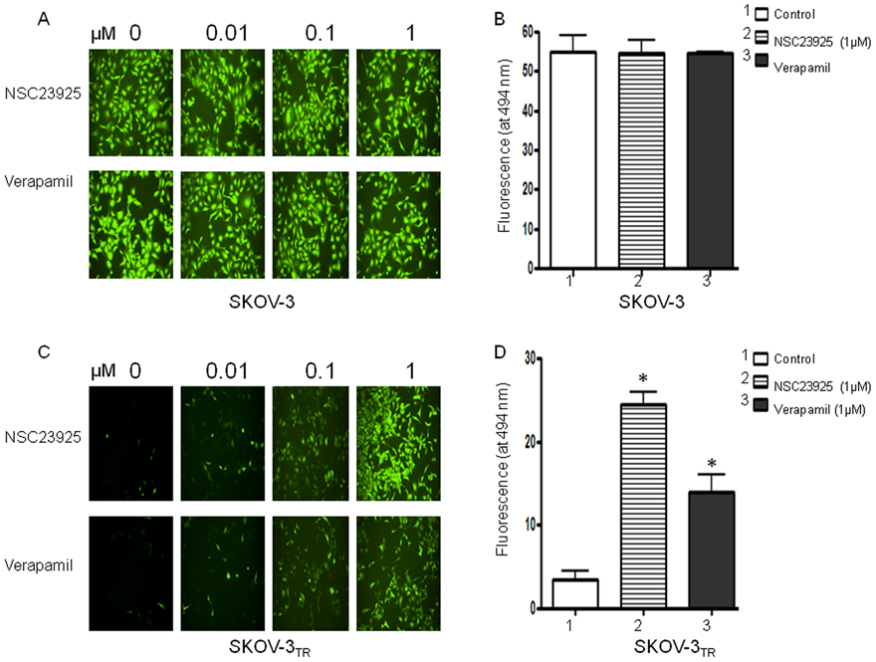
Calcein AM efflux from treated SKOV-3 and SKOV-3_TR_ cells. The Calcein-AM assay was optimized and performed using the Vybrant Multidrug Resistance kit and SKOV-3_TR_ cells. Cells were seeded at 50,000 cells/well (100 µl of culture medium) in 96-well plate and incubated for 24 h. SKOV-3_TR_ cells in triplicate were treated with NSC23925, verapamil for one hour and then incubated in calcein AM for 30 min. The cell fluorescence images were acquired by a fluorescence microscope (*A*, *C*) and quantities of fluorescence were measured in a SPECTRAmax Microplate Spectrofluorometer (*B, D*). The data were representative of one of three independent experiments. *, P<0.001.

**Figure 7:**
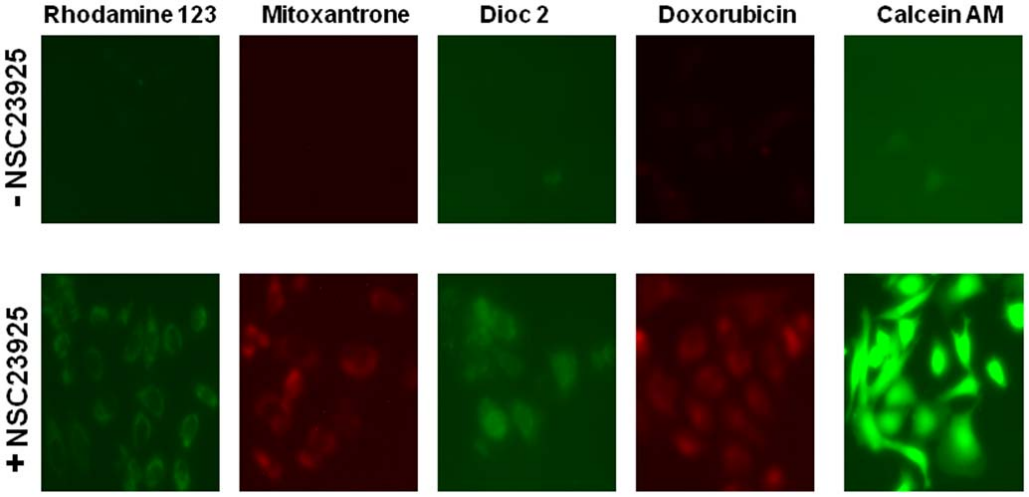
NSC23925 increases Pgp1 substrates accumulation in SKOV-3_TR_. Images of SKOV-3_TR_ cells incubated with various fluorescent substrates of Pgp1 in the presence (bottom panel) and absence (top panel) of NSC23925. For visualization of effects of NSC23925 on the intracellular retention of rhodamine 123, mitoxantrone, Dioc2, doxorobucin, and calcein AM, 10,000 resistant cells were seeded on to Lab-Tek 8-well chamber slides on the day prior to the assay. SKOV-3_TR_ cells were then incubated with either 1 µM rhodamine 123, 10 µM mitoxantrone, 0.1 µM Dioc2, 10 µM doxorubicin or 0.25 µM calcein AM either alone or in the presence of NSC23925 in RPMI 1640 media for one hour at 37°C. Image were acquired a Nikon Eclipse Ti-U fluorescence microscope (Nikon Corp.) equipped with a SPOT RT digital camera (Diagnostic Instruments, Inc., Sterling Heights, MI).

**Figure 8:**
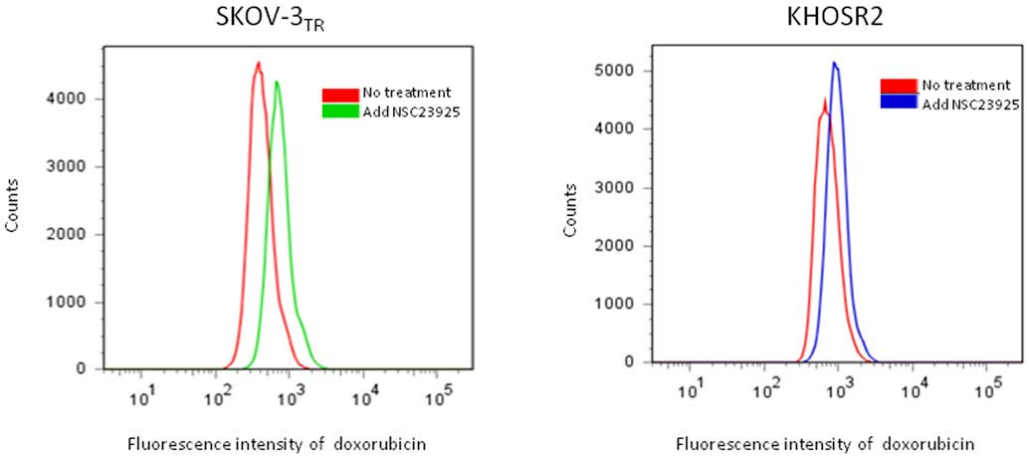
Intracellular retention of doxorubicin from multidrug resistant SKOV-3_TR_ and KHOSR2 cells in the presence of 5 µM doxorubicin with or without treatment of NSC23925 (0.5 µM). Cells were treated for 1 hour before the flow cytometry analysis.

Reversal of MDR is usually manifested as an increased intracellular accumulation of chemotherapeutics, which can be achieved by disturbing Pgp1-mediated drug uptake and efflux. Therefore, we examined the effect of NSC23925 on the uptake and efflux of several substrates of Pgp1 (calcein AM, for example) in SKOV-3_TR_ and OVCAR8_TR_. NSC23925 was shown to increase intracellular accumulation of calcein AM in these cell lines in a dose-dependent manner as determined by both image analysis (Fig. 6A and 6C) and by microplate spectrofluorometer analysis (Fig. 6B and 6D). NSC23925 has a prominent effect on the accumulation of calcein AM in these cells starting at a concentration as low as 1 nM. Half-maximal reversal of accumulation deficit was observed at 100 nM and near maximal at 500 nM. The potency of NSC23925 was significantly greater than that observed for verapamil and CsA (Fig. 6). In the control parental drug-sensitive cell lines SKOV-3 and OVCAR8, which do not overexpress Pgp1, NSC23925 had no evident effect on accumulation of calcein AM (Fig. 6A and 6B). To confirm that NSC23925 inhibition of Pgp1-mediated efflux was not specific for only calcein AM, we further evaluated NSC23925 inhibition of efflux with other Pgp substrates including Rhodamine 123, mitoxantrone, dioc 2 and doxorubicin. The fluorescence levels of these agents were monitored to determine cellular accumulation. We observed that NSC23925 inhibited the efflux of these Pgp1 substrates from SKOV-3_TR_ (Fig. 7). Flow cytometric analysis further confirmed that NSC23925 treatment increases the accumulation of doxorubicin in SKOV-3_TR_ and KHOSR2 cells (Fig. 8).

### NSC23925 stimulates Pgp1 ATPase activity, but does not affect Pgp1 expression

**Figure 9:**
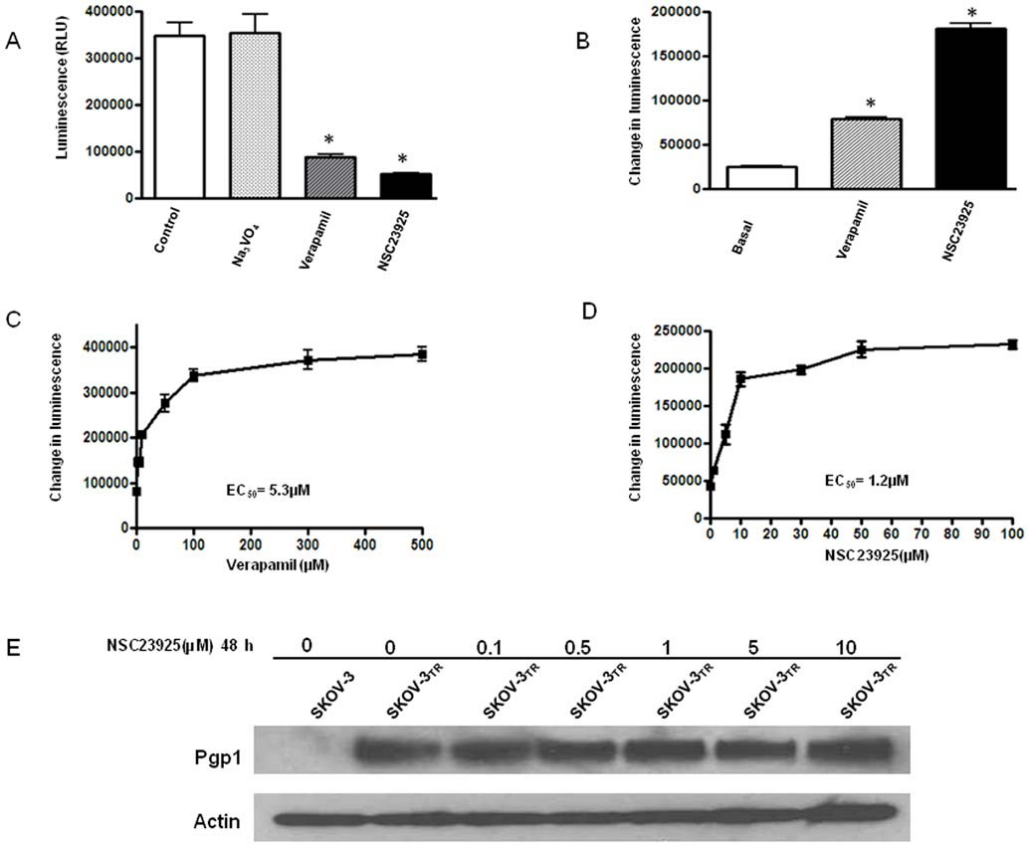
Stimulation of Pgp1 ATPase activity by NSC23925 as compared with verapamil. Untreated, 100 µM Na_3_VO_4_, 100 µM verapamil and 10 µM NSC23925 -treated Pgp1 reactions were performed according to the manufacturer's protocol. Luminescence was read on a BMG LABTECH Polarstar Optima Luminometer. The decrease in luminescence of untreated samples compared to samples plus Na_3_VO_4_ (ARLU_basal_) represents basal Pgp1 ATPase activity. The decrease in luminescence of verapamil or NSC23925 treated samples represent Pgp1 ATPase activity. *A*: Decrease in luminescence of NSC23925 treated sample as compared with Na_3_VO_4_ or Verapamil treated sample. *B*: Replotted the stimulation of Pgp1 ATPase activity by verapamil and NSC23925. *C* and *D*: Dose dependence of verapamil (*C*) and NSC23925 (*D*) stimulation of Pgp1 ATPase activity. The data were representative of one of three independent experiments. *, P<0.001. *E*: Effect of NSC23925 on expression of Pgp1 in paclitaxel resistant cells. The paclitaxel resistant cell line SKOV-3_TR_ were treated with different concentration of NSC23925 for 48 h. Equal amounts (20 µg protein) of total cell lysates were used for each sample. Pgp1 expression was determined by Western blot as described in the Materials and Methods.

The increased accumulation of intracellular drug may be a result of decreased expression of Pgp1 or decreased function of Pgp1. Pgp1 has an ATP-binding region that is essential for substrate transport, and the hydrolysis of ATP by Pgp1 ATPase is critical for restoring the transporter to its active conformational state. Thus, monitoring ATPase activity in cell membrane preparations or in purified membrane proteins allows for identification of those compounds that interact with the drug efflux transporters. Pgp1 exhibits a highly drug-dependent ATP hydrolysis activity, and a variety of Pgp1 inhibitors, as well as Pgp1 substrates, can stimulate ATPase activity [32], [33], [34], [35]. In the ATPase activity assay, a test molecule is concluded to interact specifically with Pgp1 if it significantly modulates ATPase activities, at any one of the concentrations (0.05 to 50 µM). Utilizing this method, we examined the effect of NSC23925 on both the expression and function of Pgp1. We observe that NSC23925 significantly increased the ATPase activity in purified recombinant human Pgp1 membrane protein and this stimulation was dose-dependent (Fig. 9). However, the expression level of Pgp1 was not affected by different treatments with the addition of NSC23925 (up to 10 µM for 48 h) (Fig. 9E). These results suggest that NSC23925 stimulates Pgp1 ATPase activity by directly interacting with Pgp1.

## Discussion

In this study, we screened 2,000 small molecule compounds in the NCI Structural Diversity Set to identify agents capable of reversing multidrug resistance in MDR cell lines. The present study extends the previous techniques such as flow cytometric analysis, dye efflux assay, and bioinformatic approaches to find novel MDR inhibitors by directly reversing MDR in a cell-based high-throughput assay [24], [36], [37]. The cell-based screening technique has the advantage of unbiased testing of a large number of small-molecule compounds to detect reversal of drug resistance. This approach identified a small molecular compound, NSC23925, as a novel agent that reverses MDR in Pgp1 overexpressing cell lines. NSC23925 is a novel methoxyphenyl piperidinyl compound (http://pubchem.ncbi.nlm.nih.gov/) with molecular weight 348. Unlike NSC77037/tetrandrine, information about NSC23925 pharmacological action is lacking. There is no prior report describing the action of NSC23925 on reversing chemoresistance in cancer. We demonstrated such reversal of MDR in cell lines derived from ovarian cancer, breast cancer, colon cancer, and sarcoma tumors. We observed reversal of MDR for paclitaxel, doxorubicin, ET-743, and other anti-cancer agents.

In multidrug resistant cell lines which highly express MDR1 (Pgp1 protein), Pgp1 functions as an energy-dependent membrane transporter that rapidly extrudes chemotherapeutic drugs from cells, and therefore prevents the drugs from exerting cytotoxic effects. Many studies with multidrug resistant cells correlated resistance to reduced accumulation of drugs within the cells due to increased efflux or decreased influx by Pgp1[3], [7], [10]. As Pgp1 is an ATP-dependent transport protein, agents that inhibit ATP-dependent drug transport should inhibit the efflux of drugs from resistant cells and increase intracellular accumulation. In this study, we observed that NSC23925 increased the intracellular accumulation of the Pgp1 substrates: paclitaxel, calcein-AM, rhodamine-123 (Rh-123) and doxorubicin. We show that NSC23925 stimulates ATPase activity in a dose-dependent manner, which is required for the proper function of Pgp1 [38], [39]. Although ATPase activity is closely associated with the function of Pgp1, an increase in ATPase activity does not necessarily lead to an enhancement of Pgp1 function. Previous reports have demonstrated that certain chemosensitizers enhance the ATPase activity of Pgp [33], [40], [41], [42] For example, verapamil is another example of a Pgp1 inhibitor, stimulates the ATPase activity of Pgp1, and inhibits Pgp1 function [33]. The MDR inhibitor,5,7,30,40,50-pentamethoxyflavone (PMF) stimulates the ATPase activity in a concentration dependent manner [42]. Gefitinib, an EGFR tyrosine kinase inhibitor, also stimulates ATPase activity of Pgp and directly inhibits the function of *Pgp* in multidrug resistant cancer cells [43]. Another MXR/BCRP (ABCG2) inhibitor is 6-prenylchrysin, a flavonoid compound that also inhibits MXR function by stimulating the ATPase activity of MXR [32]. However, the molecular mechanisms of enhancement of Pgp ATPase activity by these Pgp inhibitors are unknown [44], [45]. Like these Pgp or MXR inhibitors, the mechanism of how NSC23925 stimulates Pgp ATPase activity but inhibits the function of Pgp deserves further investigation.

Our core observations are that (1) the function of Pgp1 mainly functions to efflux drugs out of MDR tumor cells, (2) NSC23925 was observed to inhibit the efflux of substrates of Pgp1, such as paclitaxel and doxorubicin, (3) NSC23925 at 10 µM for 48 hours does not inhibit the expression level of Pgp1, and (4) NSC23925 stimulates Pgp ATPase activity. Taken together, these observations support the hypothesis that NSC23925 reverses MDR in tumor cells by uncoupling Pgp ATPase activity from the drug efflux activity of Pgp1.

The potency was confirmed by several assays (including cytotoxic drug sensitivity MTT assay, enhancement of drug uptake Vybrant™ multidrug resistance assay, inhibition of drug efflux assay, and mechanisms of NSC23925 inhibition by Pgp-Glo assay) using a panel of multidrug resistant cell lines with different degrees of drug resistance and Pgp1 expression. In these assays, full to partial reversal of resistance in several MDR cell lines to several major classes of chemotherapy drugs was achieved in the presence of 10 to 2,000 nM NSC23925. Direct comparison with several modulators such as verapamil and CsA in different assays demonstrated that NSC23925 is the most potent among the three modulators. NSC23925 was 20-fold more potent than verapamil and 50-fold more potent than CsA in the same concentration.

Our study shows that reversal of MDR by NSC23925 was through selective and potent inhibition of Pgp1 function. For example, in contrast to the modulatory activity in the resistant cell lines, NSC23925 had no significant effect on cytotoxic drug activity in non-Pgp1 expressing parental cell lines nor did it affect the cytotoxicity of non-Pgp1 substrates such as cisplatin and methotrexate. Moreover, the concentration of NSC23925 required to fully reverse drug resistance *in vitro* was 10 to 20 fold lower than the concentration at which any toxicity (IC_50_) was observed in the cell lines. Further confirmation of selectivity of NSC23925for Pgp1 was provided by the fact that the NSC23925, even at very high concentrations (50 µM), did not inhibit MRP1 or BCRP function. These findings provide support for further development of NSC23925 or its derivates as candidate inhibitors of MDR in cancer treatment. Such efforts can further our understanding of the molecular mechanisms of drug resistance.
